# Nanopore-targeted sequencing for rapid and accurate diagnosis of tuberculous serous effusions: a prospective evaluation across pleural, peritoneal, and pericardial fluids

**DOI:** 10.3389/fmed.2026.1826173

**Published:** 2026-04-29

**Authors:** Tianzhen Wang, Xingyu Liu, Binbin Chu, Yicheng Guo, Yi Zeng, Weiwei Gao

**Affiliations:** 1 Department of Tuberculosis, The Second Hospital of Nanjing, Affiliated to Nanjing University of Chinese Medicine, Nanjing, China; 2 Hangzhou Shengting Medical Technology Co., Ltd, Hangzhou, China; 3 Department of Tuberculosis, The Second Hospital of Nanjing, The School of Public Health of Nanjing Medical University, Nanjing, China

**Keywords:** diagnostic efficacy, drug resistance mutation, nanopore-targeted sequencing, serous effusion, tuberculosis, tuberculous pericarditis, tuberculous peritonitis, tuberculous pleural effusion

## Abstract

**Background:**

Diagnosing tuberculous serous effusions involving the pleura, peritoneum, and pericardium remains a clinical challenge, primarily due to the paucibacillary nature of such infections and the suboptimal sensitivity of conventional diagnostic modalities. This study aimed to evaluate the diagnostic performance of nanopore-targeted sequencing (NTS) for detecting the *Mycobacterium tuberculosis* complex (MTBC) in various tuberculous serous effusions, in comparison with standard diagnostic techniques. Our results demonstrated that NTS achieves significantly higher sensitivity while maintaining 100% specificity; additionally, it can simultaneously identify drug resistance-associated mutations, thereby facilitating earlier and more precise clinical decision-making for anti-tuberculosis therapy.

**Methods:**

A prospective cohort study was conducted on patients presenting with serous effusions of suspected tuberculous origin. Effusion samples (pleural, peritoneal, and pericardial) were collected via thoracentesis, paracentesis, or pericardiocentesis, respectively. All samples underwent a panel of standard tests, including acid-fast bacilli (AFB) smear microscopy, mycobacterial culture, Xpert MTB/RIF assay, and biochemical analyses (including adenosine deaminase [ADA] activity detection). The diagnostic performance of NTS was systematically evaluated and compared against both microbiological reference standards and composite clinical diagnostic criteria.

**Results:**

A total of 119 patients with serous effusions were enrolled, including 89 cases of tuberculous effusions and 30 cases of non-tuberculous effusions. The overall positive rates of AFB smear microscopy, MTB culture, and Xpert MTB/RIF assay were markedly low, at 1.12, 8.89, and 12.35%, respectively. NTS exhibited significantly superior sensitivity (61.8%) compared with other conventional microbiological tests (T-SPOT, Xpert MTB/RIF, and MTB culture; all *p* < 0.05) while retaining perfect specificity (100%). Notably, despite the limited sample sizes in the peritoneal and pericardial subgroups, NTS demonstrated notable diagnostic performance in pleural effusion samples, with preliminary findings suggesting potential utility across other serous fluid types. Furthermore, NTS provided valuable genotypic information on drug resistance mutations in MTBC-positive samples.

**Conclusion:**

NTS demonstrates substantially higher sensitivity than conventional microbiological methods for the rapid identification of tuberculous serous effusions. Its unique ability to directly detect MTBC DNA and drug resistance markers in low-bacillary-load serous fluids (pleural, peritoneal, and pericardial effusions) renders it particularly valuable for the early and precise diagnosis of tuberculous serous effusions, and it has the potential to guide the timely initiation of targeted anti-tuberculosis therapy.

## 1 Introduction

According to the 2024 WHO Global Tuberculosis Report, tuberculosis (TB), caused by *Mycobacterium tuberculosis* (MTB), has re-emerged as the leading cause of death from an infectious disease, posing a severe global public health challenge. After three years of the COVID-19 pandemic, during which SARS-CoV-2 was the primary infectious cause of mortality worldwide, TB regained its status as the top fatal infectious disease in 2023, resulting in an estimated 1.25 million deaths—nearly double the number of fatalities associated with HIV/AIDS (1).

Tuberculous serous effusions, involving the pleura, peritoneum, and pericardium, are common extrapulmonary manifestations of TB. Tuberculous pleural effusion (TPE) is the most prevalent, affecting up to 30% of TB patients in endemic areas; tuberculous peritonitis and pericarditis, though less common, are associated with significant morbidity and mortality (2, 3). Diagnosing tuberculous serous effusions is notoriously difficult in clinical practice: the paucibacillary nature of these effusions often leads to low diagnostic yields with direct smear microscopy (e.g., Ziehl-Neelsen or Auramine staining). Even mycobacterial culture, the historical gold standard for TB diagnosis, is limited by its long turnaround time (2–8 weeks) and variable sensitivity (4, 5). Automated nucleic acid amplification tests (NAATs) such as Xpert MTB/RIF and Xpert Ultra enable faster diagnosis but still exhibit suboptimal sensitivity (typically 20–50%) in serous effusion samples (6, 7). While biochemical markers including ADA and interferon-*γ* (IFN-γ) provide valuable supportive evidence for diagnosis, their specificity is compromised in clinical settings with a high prevalence of other infectious or malignant diseases (8, 9). Invasive procedures such as pleural, peritoneal, or pericardial biopsy can improve diagnostic accuracy but are not always clinically feasible due to their invasive nature, technical requirements, and the presence of patient comorbidities (10).

The World Health Organization (WHO) has emphasized the critical need for rapid, accurate, and accessible diagnostic tools for all forms of TB (11). Nanopore-targeted sequencing (NTS), a powerful third-generation sequencing technology, has emerged as a promising solution to address this unmet clinical need. Its core advantages include a rapid turnaround time (often <24 h), long-read sequencing capabilities that facilitate genomic assembly and structural variation detection, portability (e.g., the MinION device), and support for real-time data analysis. Most crucially, a single NTS assay can simultaneously identify MTBC species and detect mutations associated with resistance to first- and second-line anti-tuberculosis drugs (12). Previous studies have successfully applied NTS to TB diagnosis using sputum and bronchoalveolar lavage fluid (BALF) samples (13, 14); our research team has also validated its utility for diagnosing tuberculous meningitis using cerebrospinal fluid and for TB detection in tissue samples (9, 15). However, the comprehensive diagnostic performance of NTS for detecting MTBC in serous effusions (pleural, peritoneal, and pericardial) has not been fully elucidated.

This study aimed to systematically evaluate the diagnostic efficacy of NTS for detecting MTBC in pleural, peritoneal, and pericardial effusion samples, and to compare its performance with established conventional diagnostic methods (AFB smear microscopy, mycobacterial culture, Xpert MTB/RIF assay, and ADA testing). We hypothesized that NTS would exhibit significantly higher sensitivity than conventional methods while providing critical genotypic drug susceptibility information, making it a valuable clinical tool for the management of tuberculous serous effusions.

## 2 Materials and methods

### 2.1 Study population and ethical approval

This prospective study was conducted at the Second Hospital of Nanjing, Jiangsu Province, China, from January 2021 to October 2024. Consecutive patients with clinically suspected tuberculous serous effusions (pleural, peritoneal, or pericardial) were screened for study eligibility. The study protocol was approved by the Human Research Ethics Committee of the Second Hospital of Nanjing (Approval ID: 2024-LS-ky026). In accordance with the Declaration of Helsinki, written informed consent was obtained from all study participants or their legal guardians prior to the performance of any study-related procedures.

### 2.2 Inclusion and exclusion criteria

**Figure 1:**
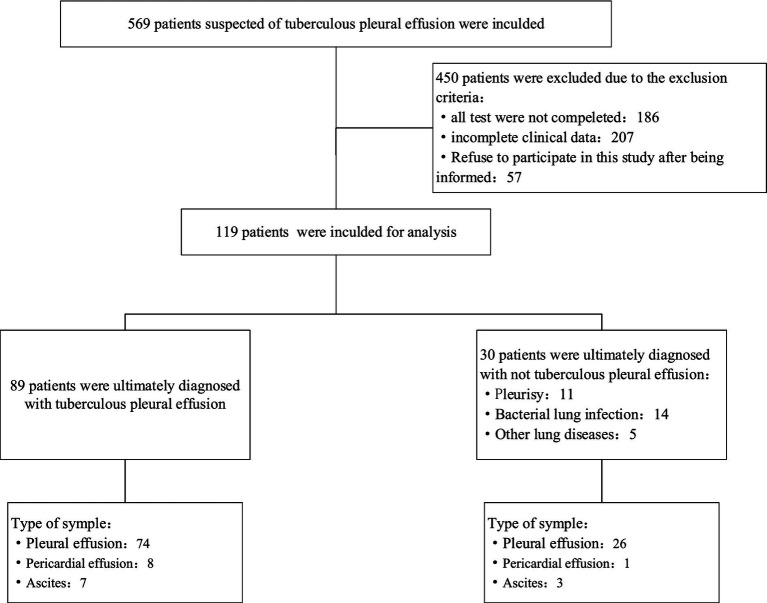
Study flowchart. Workflow of the categorization of patients involved in the study.

Patients were included if they met the following criteria: (1) Clinical and radiological (ultrasound/CT) evidence of significant pleural, peritoneal, or pericardial effusion suggestive of tuberculosis; (2) Age ≥ 16 years; (3) No contraindications to diagnostic fluid aspiration (thoracentesis, paracentesis, pericardiocentesis); (4) No anti-tuberculosis therapy (ATT) received within the past 3 months. Exclusion criteria were: (1) Contraindications to invasive procedures (e.g., bleeding diathesis, uncooperative patient); (2) Non-tuberculous effusions with a confirmed alternative diagnosis established prior to sampling (e.g., known metastatic cancer with malignant effusion confirmed cytologically on a previous tap); (3) Insufficient sample volume for complete testing. The detailed screening process is outlined in the Figure 1.

### 2.3 Sample collection and processing

Under aseptic conditions and ultrasound guidance, effusion samples (50–100 mL) were obtained via thoracentesis, paracentesis, or pericardiocentesis by experienced clinicians. The collected fluid was aliquoted immediately: 10–20 mL: Sent for routine biochemical analysis (cell count, protein, glucose, lactate dehydrogenase (LDH)) and ADA testing. 5–10 mL: Used for microbiological tests: AFB smear, liquid culture (MGIT), solid culture (Löwenstein-Jensen), and Xpert MTB/RIF Ultra assay (as per manufacturer’s instructions) 0.30–50 mL: Centrifuged (3,000 × g for 20 min); the pellet was used for AFB smear and DNA extraction for NTS and PCR. The remaining pellet and supernatant were stored at −80 °C.

### 2.4 Laboratory methods

*Routine and biochemical tests*: Cell count, protein, glucose, LDH, and ADA were performed using standard automated analyzers.

*Microbiological tests*: AFB smear was performed using Ziehl-Neelsen staining. Cultures were performed using both MGIT 960 system and Löwenstein-Jensen media following standard protocols. Xpert MTB/RIF Ultra was performed according to the manufacturer’s instructions (Cepheid).

*Nanopore-targeted sequencing (NTS)*: DNA was extracted from the centrifuged pellet using a commercial kit (QIAamp DNA Mini Kit, Qiagen). Libraries were prepared using a targeted panel designed to amplify *Mycobacterium tuberculosis* complex (MTBC)-specific genomic regions and genes associated with drug resistance, including *rpoB*, *katG*, *inhA*, *embB*, *gyrA*, *gyrB*, *rrs*, *eis*, *tlyA*, *ethA*, *Rv0678*, *atpE*, *rrl*, *rplC*, *folC*, *thyA*, *alr*, and *gidB*. Sequencing was performed on a MinION Mk1B sequencer (Oxford Nanopore Technologies) using R9.4.1 flow cells. Real-time basecalling was performed using Guppy v5.0.7 in high-accuracy mode, followed by data processing with MinKNOW software and integrated bioinformatics pipelines (EPI2ME WIMP/ARMA) for species identification and resistance mutation detection.

Raw sequencing data were filtered using the following quality control criteria: reads with a quality score < 10 were discarded; remaining reads were required to have a mapping quality ≥ 60 against the MTBC reference genome (NCBI Reference Sequence: NC_000962.3), sequence identity ≥ 90%, and a minimum length of 200 bp. Reads mapping to the human genome or common laboratory contaminants were excluded. The median sequencing depth for targeted MTBC genomic regions in NTS-positive samples was 1,012 × (interquartile range: 612–1,548×). To ensure reliable resistance mutation detection despite the higher error rate of R9.4.1 chemistry, variants were called only if supported by ≥ 50 × depth and an allele frequency ≥ 80%.

A positive NTS result was defined by the identification of at least one unique read mapping specifically to the MTBC reference genome after stringent filtering. This threshold was validated by the consistent absence of MTBC reads in negative controls (no-template control and extraction control) across all sequencing runs, and by the 100% specificity observed in 30 non-tuberculous effusion samples (Table 1).

**Table 1:** Comparison of the diagnostic performance of nts versus established diagnostic methods.

Method	Sensitivity (95% CI)	Specificity (95% CI)	PPV (95% CI)	NPV (95% CI)	AUC (95% CI)
NTS	61.8 (51.4–71.2)	100.0 (88.6–100.0)	100.0 (92.0–100.0)	46.9 (34.5–59.6)	0.809 (75.8–86.0)
T-SPOT	79.8 (70.3–86.8)	50.0 (33.2–66.8)	82.6 (73.2–89.1)	45.5 (29.8–62.0)	0.649 (54.9–74.9)
Xpert	12.4 (7.0–20.8)	93.3 (78.7–98.2)	84.6 (57.8–95.7)	26.4 (19.0–35.5)	0.528 (47.2–58.5)
MTB culture	9.0 (4.6–16.7)	93.3 (78.7–98.2)	80.0 (49.0–94.3)	25.7 (18.4–34.6)	0.512 (45.7–56.6)
AFB smear	1.1 (0.2–6.1)	100.0 (88.6–100.0)	100.0 (20.7–100.0)	25.4 (18.4–34.0)	0.506 (49.5–51.7)

*PCR*: In-house or commercial IS6110/IS1081-targeted PCR was performed on extracted DNA for comparison. The time distribution of various diagnostic test results is shown in Table 2.

**Table 2:** Time distribution of various diagnostic test results.

Diagnostic methods	Median time	Range
AFB smear	24 h	——
Cultures	18 days	12–28 days
Löwenstein-Jensen	28 days	21–42 days
Xpert MTB/RIF Ultra	3.2 h	2.5–4.0 h
NTS	22 h	18–26 h
MGIT 960 DST	11 days	7-21 days

### 2.5 Phenotypic drug susceptibility testing (DST)

For all patients with positive MTB culture isolates (either from effusion samples or other clinical specimens), phenotypic DST was performed using the MGIT 960 system (Becton Dickinson, USA) according to the manufacturer’s instructions. The following drugs were tested: streptomycin, isoniazid, rifampicin, pyrazinamide, ethambutol, and fluoroquinolones (ofloxacin or moxifloxacin). Results were interpreted using the critical concentrations recommended by the Clinical and Laboratory Standards Institute (CLSI) (16). Phenotypic DST results were used as the reference standard to validate the resistance-associated mutations detected by NTS.

### 2.6 Reference standards

*Definite TB effusion*: (a) Positive MTB culture from the effusion sample or biopsy tissue, OR (b) Positive Xpert MTB/RIF Ultra from the effusion sample with consistent clinical presentation.

*Clinical TB effusion*: Effusion with negative microbiological tests but fulfilling all of the following: (a) Lymphocyte-predominant exudate with elevated ADA (ADA ≥ 40 U/L for pleural, ADA ≥ 30 U/L for peritoneal/pericardial – adjust based on your lab’s common cut-offs or literature); (b) Clinical and radiological findings highly suggestive of TB; (c) Exclusion of other causes; (d) Favorable response to empirical anti-tuberculosis therapy during follow-up.

To minimize incorporation bias, diagnostic performance was evaluated separately against the microbiological reference standard (definite TB) and the composite clinical reference standard (definite TB + clinical TB).

*Non-TB effusion*: Effusion with a confirmed alternative diagnosis (e.g., positive cytology for malignancy, positive culture for other pathogens, or definite diagnosis of another inflammatory condition like lupus) OR resolution without anti-TB treatment during follow-up.

### 2.7 Statistical analysis

Statistical analysis was performed using SPSS software (version 27.0) and R 4.4.3. For each diagnostic test (AFB smear, MTB culture, Xpert Ultra, NTS), sensitivity, specificity, positive predictive value (PPV), and negative predictive value (NPV) were calculated against the reference standard (definite TB for microbiological tests; definite + clinical TB vs. non-TB for overall performance). The 95% confidence intervals (CIs) for these proportions were estimated using the Wilson score method. The McNemar test was used to compare the sensitivities of NTS with those of other tests among the TB patients (paired design). A subgroup analysis was planned *a priori* to assess NTS performance across different effusion types (pleural, peritoneal, pericardial). For comparisons between independent groups (e.g., pleural vs. pericardial effusion) with small sample sizes, Fisher’s exact test was applied. Due to the limited number of cases in the peritoneal and pericardial subgroups, no statistical comparisons were performed for paired tests within these subgroups; only descriptive statistics are reported. A *p*-value of < 0.05 was considered statistically significant.

## 3 Results

### 3.1 Patient characteristics

This study included 119 patients, 89 of whom were diagnosed with TB and 30 with non TB. The basic demographic characteristics and clinical data of the patients are summarized in Table 3.

**Table 3:** Baseline demographic and clinical characteristics of the study participants.

Characteristic	ALL (*n* = 119)	TB (*n* = 89)	Non-TB (*n* = 30)	*p*-value
Age Median[IQR]	54.0 [33.0]	56.0 [31.0]	50.0 [41.0]	0.249
Gender (*n*, %)				0.007
Male	84 (70.6)	69 (77.5)	15 (50.0)	
Female	35 (29.4)	20 (22.5)	15 (50.0)	
Underlying condition				0.496
Yes	60 (50.4)	47 (52.8)	13 (43.3)	
No	59 (49.6)	42 (47.2)	17 (56.7)	
Diabetes				0.549
Yes	19 (16.0)	13 (14.6)	6 (20.0)	
No	100 (84.0)	76 (84.5)	24 (80.0)	
Hypertension				0.234
Yes	22 (18.5)	20 (22.5)	2 (6.7)	
No	97 (81.5)	69 (77.5)	28 (93.3)	
Cardiovascular disease				0.234
Yes	15 (12.6)	13 (14.6)	2 (6.7)	
No	104 (87.4)	76 (84.5)	28 (93.3)	
Tumor				0.572
Yes	4 (3.4)	4 (4.5)	0 (0.0)	
No	115 (96.6)	85 (95.5)	30 (100.0)	
Autoimmune disease				0.030
Yes	13 (10.9)	7 (7.9)	6 (20.0)	
No	106 (89.1)	82 (92.1)	24 (80.0)	
Total cell count of pleural effusion	2.1 [3.3]	2.02 [2.85]	2.70 [3.99]	0.068
Biochemical indicators				
Total protein	44.2 [11.1]	44.5 [10.9]	42.50 [11.75]	0.054
Albumin	27.0 [9.0]	27.9 [9.6]	26.20 [9.25]	0.113
LDH	442.0 [469.0]	420.0 [443.0]	535.0 [562.5]	0.245
Suger	5.6 [2.6]	5.7 [2.7]	4.5 [2.3]	<0.01
ADA	42.0 [42.0]	54.0 [54.0]	32.0 [14.3]	<0.01
Chloride	105.0 [8.0]	107.0 [5.5]	97.5 [46.5]	0.006
Sample type				0.557
Ascites	10 (8.4)	7 (7.9)	3 (10.0)	
Pericardial effusion	9 (7.6)	8 (9.0)	1 (3.3)	
Pleural effusion	100 (84.0)	74 (83.1)	26 (86.7)	
IFN-γ Median [IQR]	376.0 [816.7]	541.5 [657.8]	3.8 [18.8]	<0.01

Demographic analysis shows that the median age of all patients is 54.0 years old. Males comprised 70.6% (84/119) of the total population, and their proportion in the TB group is significantly higher than that in the non TB group. In terms of complications, hypertension, diabetes and cardiovascular diseases are more common in TB patients.

There were no statistically significant differences in total protein, lactate dehydrogenase, and total cell count between the TB group and the non TB group in the Light’s criteria related indicators of pleural effusion. However, other biochemical indicators showed that the levels of adenosine deaminase (ADA) and interferon-*γ* (IFN-γ) in the TB group were significantly higher than those in the non TB group. In addition, the glucose level was higher and the chloride level was significantly increased in the pleural effusion of the TB group. Detailed baseline characteristics are presented in Table 3.

### 3.2 Diagnostic performance of conventional tests

**Figure 2:**
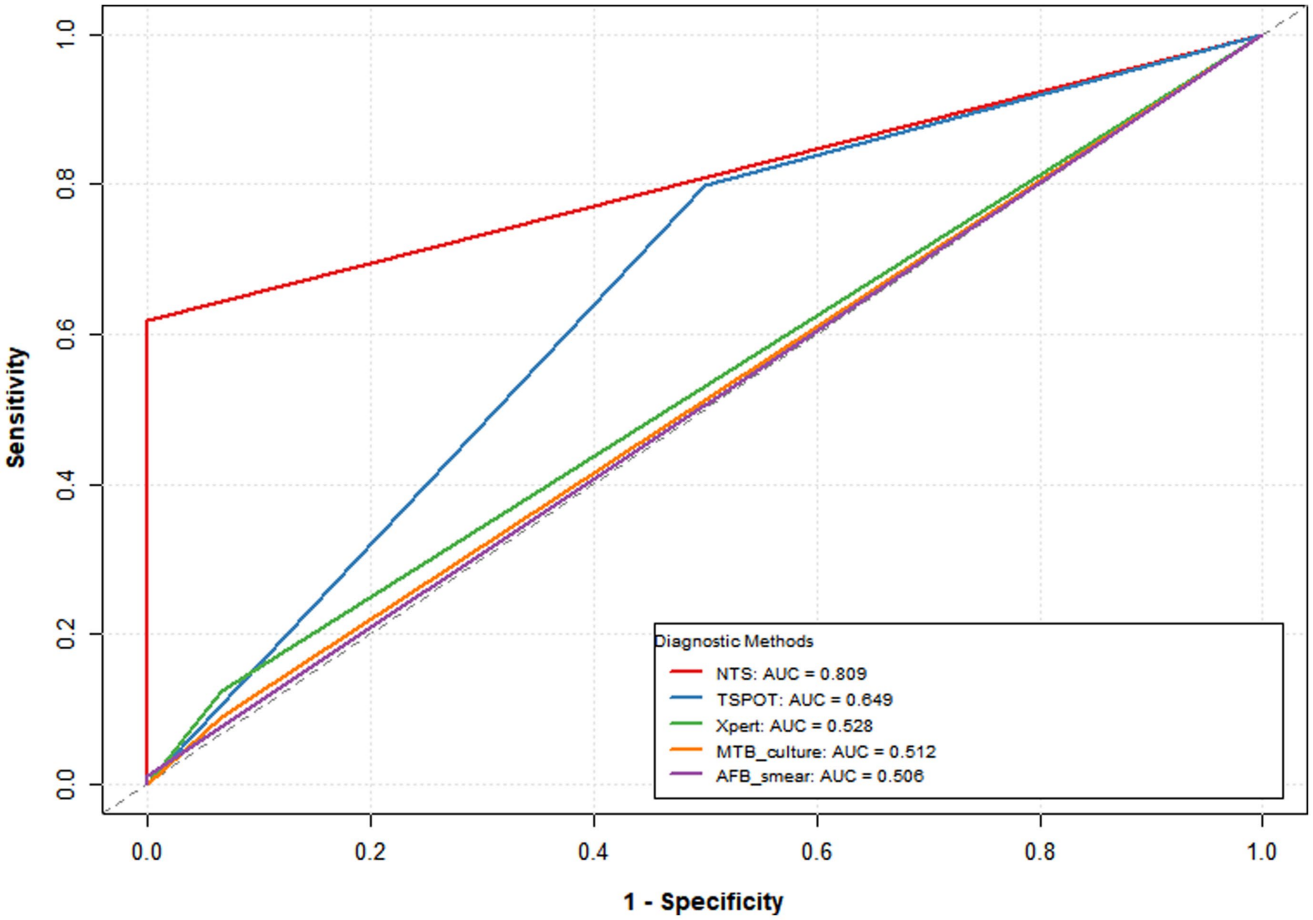
ROC curves of the different diagnostic methods.

This study compared the diagnostic performance of Nanopore Targeted Sequencing (NTS) with conventional detection methods, including acid-fast bacillus smear, TSPOT, Xpert, and mycobacterial culture. As shown in Table 1 and Figure 2, among various tests, both NTS and AFB smear exhibited the best specificity (both 100.0%; 95% CI: 88.6–100.0), but AFB smear had the lowest sensitivity, only 1.1%. Meanwhile, NTS demonstrated the best performance in terms of the Area Under the Receiver Operating Characteristic Curve (AUC), with a value of 0.809 (95% CI: 75.8–86.0). Although TSPOT had higher sensitivity (79.8%), its specificity was significantly lower (50.0%).

**Figure 3:**
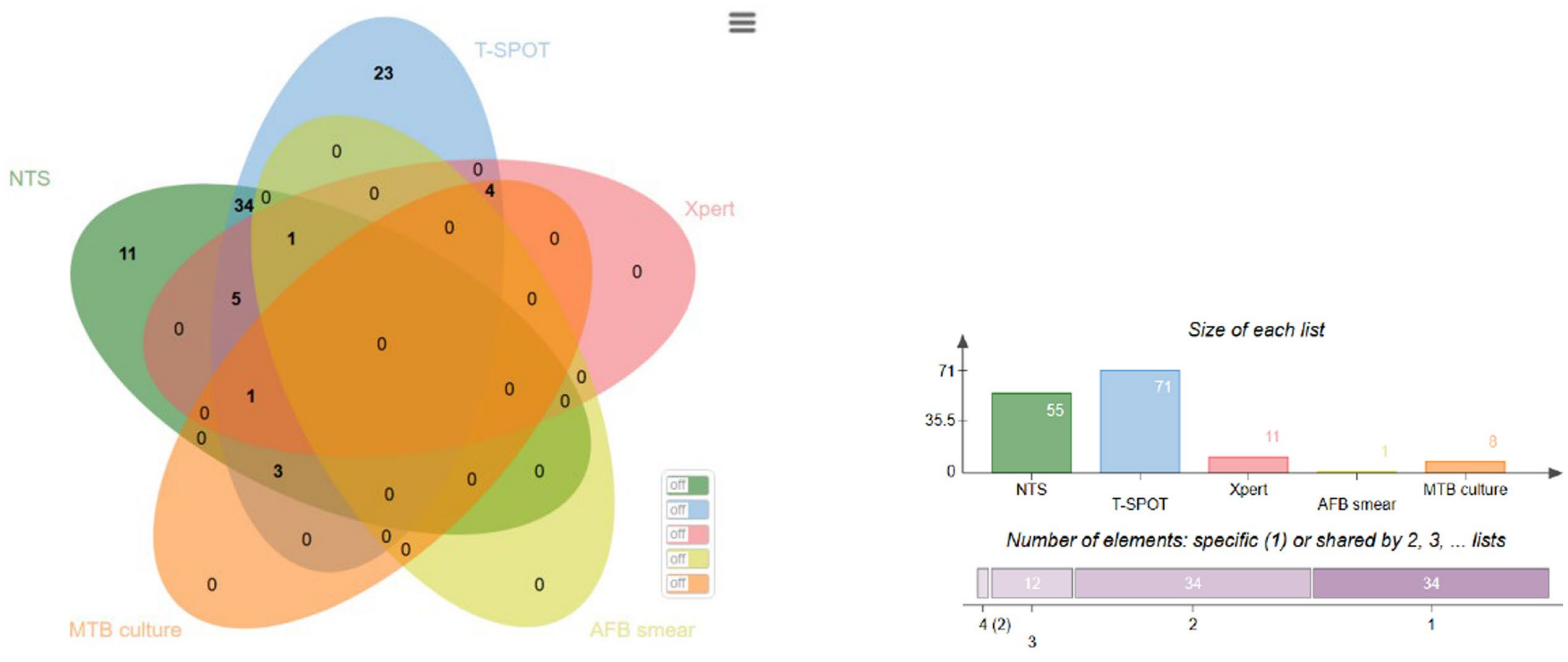
Venn diagram of positive tests for tuberculosis. Bardou et al. (18). License: Creative Commons Attribution 4.0 International License (CC BY 4.0). Source: The diagram was generated using the online tool “jvenn”, provided by GenoToul Bioinformatics platform, INRAE Toulouse. Available at: https://jvenn.toulouse.inrae.fr/app/example.html.

As shown in the Venn diagram in Figure 3, we compared the positive detection rates of five detection methods (NTS, T-SPOT, Xpert MTB/RIF, MTB culture, AFB smear) in 89 patients with tuberculous serous effusion. T-SPOT detected the most positive cases (71 cases), followed by NTS (55 cases). 34 patients were detected as positive by both NTS and T-SPOT simultaneously; Another 34 patients were detected by only one method alone (11 positive for NTS alone and 23 positive for T-SPOT alone). In addition, a few cases were detected by a combination of three or four methods: 5 cases were positive for NTS, T-SPOT, and Xpert; Three cases were positive for the combination of NTS, T-SPOT, and MTB cultures; Four cases were positive for the combination of T-SPOT, Xpert, and MTB cultures; There were no cases where all five methods were positive, but two patients were detected positive by all four methods simultaneously (NTS, T-SPOT, Xpert combined with MTB culture or AFB smear, respectively). This result suggests that NTS and T-SPOT have complementary detection capabilities, and NTS maintains high sensitivity without false positives, demonstrating more reliable diagnostic characteristics.

### 3.3 Subgroup analysis: diagnostic performance of NTS in different types of serous effusion

In all 89 cases of tuberculous serous effusion, the overall sensitivity of NTS was 61.8% (55/89; 95% CI: 51.4–71.2). Compared with traditional pathogen detection methods, the sensitivity of NTS is significantly higher than Xpert MTB/RIF (12.4%, *p* < 0.001) and MTB culture (9.0%, *p* < 0.001). The sensitivity of AFB smear is extremely low (1.1%), although the value is much lower than NTS, due to the small number of positive cases, no statistical difference was shown (*p* = 1.000). Although T-SPOT showed higher sensitivity (79.8%, *p* = 0.024), its specificity was lower (50.0%). As shown in the Table 1.

In the analysis by fluid type, NTS demonstrated notable detection ability in pleural effusion (*n* = 74). For ascites (*n* = 7) and pericardial effusion (*n* = 8), preliminary findings suggest potential utility, as detailed in Table 4. However, due to the small number of tuberculosis cases in these subgroups, statistical comparisons were not performed, and the results should be interpreted as exploratory.

**Table 4:** Sensitivity of different diagnostic methods in tuberculous serous effusion (overall and stratified by sample type).

Sample type	Method	Sensitivity (95% CI)	Positive cases (*n*/*N*)
Overall (*N* = 89)	NTS	61.8 (51.4–71.2)	55/89
	T-SPOT	79.8 (70.3–86.8)	71/89
	Xpert MTB/RIF	12.4 (7.0–20.8)	11/89
	MTB Culture	9.0 (4.6–16.7)	8/89
	AFB Smear	1.1 (0.2–6.1)	1/89
Pleural effusion (*n* = 74)	NTS	63.5 (51.5–74.4)	47/74
Ascites (*n* = 7)	NTS	57.1 (18.4–90.1)	4/7
Pericardial effusion (*n* = 8)	NTS	50.0 (15.7–84.3)	4/8

### 3.4 Drug resistance mutation detection results

Among the samples diagnosed with tuberculous effusion (Definite TB), NTS successfully identified 5 cases (5/89, 5.61%) with drug resistance related gene mutations. The specific resistance spectrum is shown in Table 5: one case was only resistant to streptomycin; 1 case of resistance to isoniazid and streptomycin; 1 case of resistance to rifampicin and streptomycin; One case of multiple resistance to rifampicin, isoniazid, and streptomycin; Another case showed widespread resistance to streptomycin, isoniazid, rifampicin, pyrazinamide, ethambutol, and fluoroquinolones. In this small subset of samples with resistance, mutations conferring resistance to streptomycin were most frequently identified (5/5), followed by isoniazid (3/5) and rifampicin (3/5).

**Table 5:** Genotypic drug resistance profiles detected by nanopore-targeted sequencing.

Patient	Sample type	Drug resistant general
1	Pleural effusion	Streptomycin
2	Pleural effusion	Streptomycin, isoniazid
3	Pleural effusion	Streptomycin, rifampicin, isoniazid
4	Pericardial effusion	Streptomycin, rifampicin
5	Ascites	Streptomycin, isoniazid, rifampicin, pyrazinamide, ethambutol, fluoroquinolones

All five drug-resistant cases were independently validated using phenotypic drug susceptibility testing (DST) performed with the MGIT 960 system. The results demonstrated 100% concordance between the NTS-detected resistance-associated mutations and the phenotypic DST profiles, confirming the reliability of the NTS findings, albeit in a small subset of samples. We acknowledge that the limited number of drug-resistant cases (*n* = 5) restricts the statistical power for evaluating the accuracy of NTS-based resistance profiling. Consequently, these results should be considered preliminary, and larger prospective studies are required to further validate the performance of NTS for drug resistance detection in tuberculous serous effusions. Importantly, the rapid availability of these resistance profiles enables prompt tailoring of anti-tuberculosis therapy, which was particularly critical for the patient with extensive drug resistance.

The sequencing depth and coverage of drug resistance-associated genes were assessed for all NTS-positive samples. The median depth across all targeted resistance loci was 1,012 × (range: 412–2,216×), with >99% of targeted amplicons achieving ≥ 100 × depth. Although the R9.4.1 flow cell chemistry has a higher raw error rate, our stringent quality control (Q-score ≥ 10, mapping quality ≥ 60, identity ≥ 90%) enabled high-confidence variant calling. The reliability of this approach was supported by the 100% concordance between NTS-detected resistance mutations and phenotypic DST results in all five drug-resistant cases.

## 4 Discussion

This study comprehensively evaluates the application of Nanopore-targeted sequencing (NTS) for the diagnosis of tuberculous serous effusions, encompassing pleural, peritoneal, and pericardial fluids. Our key finding is that NTS offers improved diagnostic sensitivity compared with conventional microbiological methods, significantly outperforming conventional microbiological methods like smear microscopy, culture, and the Xpert MTB/RIF Ultra assay. The high AUC (0.809) observed in our cohort highlight the potential of NTS to become a valuable complementary tool for diagnosing these challenging paucibacillary infections.

The low sensitivities of AFB smear (1.1%) and MTB culture (9.0%) in our study align with the well-established literature on the pauci-bacillary nature of tuberculous serous effusions (4, 5). While NAATs like Xpert Ultra represent a major advance, their sensitivity in effusions (12.4% in our study, often reported up to 50% (6, 7)) remains suboptimal for ruling out TB. NTS, with its deep sequencing capability and minimal background noise through targeted enrichment, overcomes the limitation of low bacterial load more effectively than amplification-based point tests like Xpert. Furthermore, the ability of NTS to simultaneously provide comprehensive information on drug resistance is a monumental advantage over other rapid tests, enabling informed initiation of appropriate therapy much earlier than is possible with phenotypic drug susceptibility testing (DST), which requires culture growth.

The notable diagnostic performance of NTS in pleural effusion samples, coupled with preliminary findings in peritoneal and pericardial effusions, suggests its potential applicability for extrapulmonary tuberculosis diagnostics. However, the small sample sizes for peritoneal (*n* = 7) and pericardial (*n* = 8) effusions limit the statistical robustness of cross-fluid type comparisons; therefore, these findings should be considered exploratory and warrant validation in larger, dedicated cohorts. Notably, the negative predictive value (NPV) of 46.9% remains clinically relevant—particularly given that peritoneal and pericardial tuberculosis are often even more challenging to diagnose than pleural tuberculosis—as it may help avoid unnecessary empirical anti-tuberculosis treatment in a substantial proportion of patients.

The operational characteristics of NTS, with a median turnaround time of 22 h, enable both diagnostic confirmation and drug resistance profiling within a single day. This represents a substantial improvement over culture-based methods, which require weeks to yield results. Although Xpert MTB/RIF Ultra provides faster results (approximately 3 h), it does not offer the comprehensive resistance information that NTS can deliver. In the context of tuberculous serous effusions—where timely and appropriate therapy is critical—the ability to obtain actionable information within 24 h may facilitate earlier initiation of targeted treatment and reduce unnecessary empirical therapy.

Our findings are supported by a growing body of evidence on the use of sequencing technologies for TB diagnosis (9, 12, 13, 15). The July 2025 publication by Yu Chen et al. on Nanopore Sequencing for Tuberculous Serous Effusions signals a shift in research focus toward this application (17). The real-time nature, decreasing costs, and portability of Nanopore technology make NTS a promising candidate for implementation in central laboratories in high-TB-burden countries.

Although NTS demonstrated excellent diagnostic performance in this study, exploring the synergistic effect of T-SPOT is of practical significance considering its widespread clinical application and higher sensitivity. Our data supports a potential optimized diagnostic pathway: T-SPOT can be used as a first-line high-sensitivity screening tool for patients suspected of having tuberculous serous effusion. For cases where T-SPOT is positive but routine microbiological examination is negative, especially when clinical decision-making (such as whether to initiate treatment or perform invasive biopsy) is difficult, NTS can serve as a powerful supplementary validation tool. NTS can not only confirm the presence of pathogens, but also provide critical resistance information, achieving a seamless transition from “screening” to “precise diagnosis and treatment guidance,” which is expected to shorten diagnostic delays and reduce unnecessary empirical treatment or invasive procedures.

### 4.1 Limitations

Our study has certain limitations. First, this was a single-center study, which may limit the generalizability of our findings. Second, the numbers of peritoneal (*n* = 7) and pericardial (*n* = 8) tuberculosis cases were small, restricting the statistical power of subgroup analyses for these specific effusion types; therefore, our observations in these subgroups should be considered exploratory and require validation in larger, multi-center cohorts. Third, the use of composite clinical criteria (including treatment response) as part of the reference standard may have introduced some incorporation bias, potentially leading to a modest overestimation of diagnostic performance. Fourth, the cost-effectiveness of implementing NTS compared with existing algorithms (e.g., ADA combined with Xpert) in different healthcare settings remains to be evaluated. Fifth, the technical expertise required for library preparation and bioinformatics analysis currently poses a barrier to widespread implementation, although continuous improvements in automated analysis pipelines are mitigating this challenge. Finally, only five drug-resistant cases were detected in this study, which precludes a definitive assessment of NTS’s sensitivity and specificity for resistance mutation detection. While all five NTS-detected resistance mutations were 100% concordant with phenotypic DST (MGIT 960), we did not perform line probe assays (e.g., GenoType MTBDRplus) or whole-genome sequencing for cross-validation. Future studies with larger numbers of drug-resistant tuberculous serous effusions are warranted to fully benchmark NTS against both phenotypic and genotypic reference methods.

## 5 Conclusion

In conclusion, Nanopore-targeted sequencing represents a valuable diagnostic tool for tuberculous serous effusions. Its high sensitivity and specificity across pleural, peritoneal, and pericardial fluids, coupled with its rapid turnaround time and ability to detect drug resistance, address critical gaps in the current diagnostic landscape for extrapulmonary TB. We recommend the integration of NTS into the diagnostic workflow for suspected tuberculous serous effusions, particularly in cases where conventional methods yield negative results but clinical suspicion remains high. Further efforts should focus on streamlining laboratory protocols, reducing costs, and validating its impact on patient outcomes in diverse clinical settings.

## Data Availability

The original contributions presented in the study are publicly available. This data can be found here: https://ngdc.cncb.ac.cn/gsa/browse/CRA041733, CRAO41733.
